# Re-evaluation of histological diagnoses of malignant mesothelioma by immunohistochemistry

**DOI:** 10.1186/1746-1596-5-47

**Published:** 2010-07-06

**Authors:** Helmut P Sandeck, Oluf D Røe, Kristina Kjærheim, Helena Willén, Erik Larsson

**Affiliations:** 1 Department of Pathology and Medical Genetics, St. Olav University Hospital, Erling Skjalgssons gt. 1, N-7006 Trondheim, Norway; 2 Department of Oncology, St. Olav University Hospital, Olav Kyrres gt. 17, N-7006 Trondheim, Norway; 3 Cancer Registry of Norway, Postboks 5313 Majorstuen, N-0304 Oslo, Norway; 4 Department of Clinical Pathology and Cytology, Uppsala University Hospital, Rudbeck Laboratory, Dag Hammarskjölds väg 21, S-751 85 Uppsala, Sweden; 5 Department of Genetics and Pathology, Uppsala University, Rudbeck Laboratory, Dag Hammarskjölds väg 21, S-751 85 Uppsala, Sweden; 6 Department of Laboratory Medicine, Children's and Women's Health, Norwegian University of Science and Technology, Trondheim, Norway

## Abstract

**Background:**

In order to provide reliable tissue material for malignant mesothelioma (MM) studies, we re-evaluated biopsies and autopsy material from 61 patients with a diagnosis of MM from the period of 1980-2002.

**Methods:**

Basic positive (Calretinin, EMA, Podoplanin, Mesothelin) and negative (CEA, Ber-Ep4) immunohistochemical (IHC) marker reactions were determined. If needed, more markers were used. Histological diagnoses were made by three pathologists. Survival data were calculated.

**Results:**

49 cases (80%) were considered being MM by a high degree of likelihood, five more cases possible MM. Of the remaining seven cases, three were diagnosed as adenocarcinoma, three as pleomorphic lung carcinoma, in one peritoneal case a clear entity diagnosis could not be given. One of the possible MM cases and two of the lung carcinoma cases had this already as primary diagnoses, but were registered as MM.

With a sensitivity of 100%, Calretinin and CEA were the most reliable single markers. The amount of MM cells with positive immunoreactivity (IR) for Podoplanin and Mesothelin showed most reliable inverse relation to the degree of atypia.

In the confirmed MM cases, there had been applied either no IHC or between one and 18 markers.

The cases not confirmed by us had either lacked IHC (n = 1), non-specific markers were used (n = 4), IR was different (n = 1), or specific markers had not shown positive IR in the right part of the tumour cells (n = 3).

46 of the 49 confirmed and three of the not confirmed cases had been diagnosed by us as most likely MM before IHC was carried out.

**Conclusions:**

In order to use archival tissue material with an earlier MM diagnosis for studies, histopathological re-evaluation is important. In possible sarcomatous MM cases without any positive IR for positive MM markers, radiology and clinical picture are essential parts of diagnostics. IHC based on a panel of two positive and two negative MM markers has to be adapted to the differential diagnostic needs in each single case. New diagnostic tools and techniques are desirable for cases where IHC and other established methods cannot provide a clear entity diagnosis, and in order to improve MM treatment.

## Background

Modern molecular techniques applied on tissue specimens have increased the interest in using archival material for research purposes. The risk of wrong diagnosis by doing so might be high due to the lack of immunohistochemical (IHC) evaluation at the time of diagnosis.

Malignant mesothelioma (MM) is a tumour that traditionally has been considered being difficult to diagnose histologically in a substantial subset of cases. To our knowledge, the extent of misdiagnosis of MM in archival material has not been studied systematically earlier, with the exception of one recent study [1].

MM is a highly aggressive tumour defined as derived from mesothelial cells [2,3]. It is considered an almost incurable disease. Its molecular profile indicates that most of the known genes for radio- and chemoresistance are overexpressed [4,5].

MM has traditionally been divided into epithelial, sarcomatous and mixed types, where the less common mixed and sarcomatous variants show a poorer survival. Sometimes a desmoplastic type is added, which may be diagnosed as either epithelial, mixed or sarcomatous type [6,7]. For the mixed type MM, International Mesothelioma Panel (IMP)/World Health Organization (WHO) require the presence of arbitrarily at least 10% of either an epithelial or a sarcomatous component [3]. According to recent studies, median survival after diagnosis is 4.5 to 17 months, depending on histological type, tumour stage, performance status and treatment, and other factors as gender and age [8-11].

In Norway, registered new MM cases have continuously increased from five cases in the period of 1958-62 to 382 cases in the period of 2003-07. In the last period, 88% of registered cases were histologically verified. By adding the cytologically diagnosed cases the rate of morphologically diagnosed cases reached nearly 100% (97.5% of 360 cases in the period of 2001-05) [12-14]. However, in its recent guidelines the European Respiratory Society (ERS)/European Society of Thoracic Surgeons (ESTS) Task Force does not recommend making a MM diagnosis based on cytology alone because of the high risk of diagnostic error [15].

Before IHC became a part of routine pathology in the end of the 1980s/beginning of the 1990s, and especially before more MM-specific positive IHC markers were introduced a few years later, it could be difficult or even impossible to differentiate MM from other malignant tumours, such as primary or metastatic adenocarcinomas (AC) in a part of the cases. Around 1995, antibodies against Wilms tumour protein (WT) 1, around 1998, against Calretinin and around 2005, against Podoplanin/D2-40 were introduced into routine diagnostics as new positive MM markers [16-22].

The introduction of such more specific positive markers, in combination with distinctive negative markers has significantly improved the possible accuracy of MM diagnosis. However, there is no reliable single MM IHC marker. The IMP/WHO and the International Mesothelioma Interest Group (IMIG) recommendations on MM IHC diagnostics comprise a panel of at least two positive and two negative antibodies [3,23].

This study aimed primarily at establishing histopathological diagnoses on archival tissue material from patients diagnosed as MM, in order to get a reliable basis for further studies using recent histological and IHC criteria. Furthermore, we wished to find out how the availability and choice of IHC markers may have influenced MM diagnosis.

## Methods

The archival material in this study has been used in two previous studies where verification of the histological MM diagnoses was mandatory [11,24]. Use of the material was approved by the Regional Ethical Committee.

Paraffin-embedded tissue material from 73 patients taken between 1980 and 2002 was received from 19 Norwegian departments of pathology. Primary MM diagnoses had been given mostly based on tumour biopsies, and/or in a few cases on autopsy material. Tissue specimen volumes varied from a few mm^3 ^in needle biopsies to some cm^3 ^in autopsy material. Clinical and radiological data were fragmentary, and limited to those received in written form by the primarily diagnosing pathologists in the histology requisitions. After having received additional clinical information, tumour localisation was adjusted to both pleura and peritoneum in three cases, and to scrotum in one case, compared with earlier data [11,24]. From two of the four patients with MM both in pleura and peritoneum, autopsy material from both localisations was disposable, from the others the biopsies were only from one site.

From the paraffin blocks, Hematoxylin-Eosin-Saffron (HES) stained slides were made. For 61 patients (84%), the samples showed a sufficient amount of remaining tumour tissue suitable for diagnosis by the planned IHC procedure. Preliminary, exclusively HES-based tentative diagnoses were established. Basic histological parameters were determined on HES-stained slides. Mitoses were counted per so-called high-power fields (HPF), constituted by 40 times of objective and 10 times of ocular magnification.

After having determined optimal antibody dilution individually for each marker, by applying established IHC protocols from routine pathology and in the remaining cases by testing optimal dilutions, IHC was performed by the standardised Dako Cytomation protocol DAKO REAL™ EnVision™ with peroxidase/diaminobencidine and chromogen, rabbit/mouse [25]. The minimum procedure included four positive (Calretinin, Epithelial Membrane Antigen (EMA) membranous, Mesothelin, Podoplanin) and two negative (carcino-embryonic antigen (CEA), Ber-Ep4) markers, and in a subset of cases, if considered necessary, additional IHC markers (each case including, as a minimum, Hector Battifora Mesothelial Cells (HBME) 1 and Thrombomodulin as positive and CD15 and Sialosyl-TN as negative markers, before applying Mesothelin and Podoplanin) and Alcian Blue staining. In order to reach a reliable MM diagnosis, in five cases of epithelial type MM (No. 30, 39, 62, 74, 75) (12%) and in two cases of mixed MM (nr. 11 and 25) (25%), this second set of four basic IHC markers was applied. Reasons for this were too weak and/or not typical immunoreactivity (IR).

Slides were evaluated in multiple steps defined by the used basic and additional antibodies, by two pathologists (E. Larsson, H. Sandeck) independently, finally by a third pathologist (H. Willén), before establishing a diagnosis without access to the previous histological diagnoses. After each step, the diagnosis or eventual markers to add were determined by consensus. Our MM selection criteria included the histological MM variants and subtypes according to the current WHO MM classification and clearly positive IHC staining in at least a part of the tumour tissue, or absence of staining by the mentioned positive and negative markers, respectively [3]. For the diagnosis of a sarcomatous type or component, we regarded a predominant spindle cell pattern as indispensable [26]. A definitive entity diagnosis was only given when there was a high degree of likelihood for the entity based on morphology and IHC, with positive IR for at least one positive MM marker (Table 1).

**Table 1:** Immunohistochemical markers used for malignant mesothelioma diagnosis in this study

IHC marker	Antigen type	Clone	Producer	Code No.	Dilution
**Positive markers**					
Calretinin	Calcium-binding protein	polyclonal	Zymed	18-0211	1: 1,500
EMA	HMFG protein	E-29	Dako	M0613	1: 750
Thrombomodulin	endothelial cell transmembrane glycoprotein	1009	Dako	M0617	1: 50
HBME-1	mesothelial cell membrane protein	HBME-1	Dako	M3505	1: 50
Mesothelin	cell surface glycoprotein	5B2	Novocastra	NCL-L-MESO	1: 10
Podoplanin	transmembrane mucoprotein	n.s., monoclonal	Angiobio	11-003	1: 50
CK5/6	IMF	D5/16 B4	Dako	M7237	1: 80
CK7	IMF	OV-TL12/30	Dako	M7018	1: 1,000
CK AE1/AE3	IMF (pan-CK cocktail, subfamilies A and B)	AE1 and AE3	Dako	N1590	1: 75
CK KL1	IMF (pan-CK cocktail)	KL1	Serotec	MCA144H	1: 50
**Negative markers**					
monoclonal CEA	Gold 1 epitope	II-7	Dako	M7072	1: 200
polyclonal CEA	CEA and CEA-like proteins	polyclonal	Dako	A0115	1: 13,000
Ber-Ep4	transmembrane glycoprotein	Ber-Ep4	Dako	M 0804	1: 200
CD15	Lewis X carbohydrate antigen	C3D-1	Dako	M0733	1: 25
Sialyl-TN	Sialosyl-Tn1 glycoprotein	HB-STn1	Dako	M0899	1: 100
TTF-1	nuclear transcription factor	8G7G3/1	Dako	M3575	1: 100
**Others**					
CK20	IMF	Ks 20.8	Dako	M7019	1: 200
CD10	neutral endopeptidase 24.11	SS2/36	Dako	M0727	1: 50
CD34	single-chain transmembrane protein	QBEnd 10	Dako	M7165	1: 100
CD68	glycosylated lysosomal membrane protein	KP1	Dako	M814	1: 3,000
CD99	MIC2 gene products	12E7	Dako	M3601	1: 100
Bcl-2	apoptosis inhibitor	124	Dako	M0887	1: 200
SMA	microfilaments	1A4	Dako	M0851	1: 300
Desmin	IMF	D33	Dako	M0760	1: 100
S-100	Calcium-binding proteins	polyclonal	Dako	Z0311	1: 3,000
HMB-45	part of neuraminidase-sensitive glycoconjugate in melanosomes	HMB45	Dako	M0634	1: 100
HHF-35	muscle actin	HHF 35	Dako	M0635	1: 200
MIB1	nuclear protein, proliferation marker	Ki-67/MIB-1	Dako	M7240	1: 200

Abbreviations:

IMF - intermediate filament; EMA - Epithelial Membrane Antigen; HMFG - Human Milk Fat Globule; HBME - Hector Battifora Mesothelial (Human Mesothelial Cell Antigen); CK - Cytokeratin; AE - Anti-Epithelial; KL1 - Keratin L1; CD - Cluster of Differentiation; TTF - Thyroid Transcription Factor; Bcl - B-cell lymphoma; SMA - Smooth Muscle Actin; HMB - Human Melanoma Black; HHF - Human Heart Fibroblasts/myofibroblasts; MIB - Molecular Immunology Borstel; Ki - Kiel

Cases with negative IR were coded 0 and those with positive IR were classified into quartiles (H. Sandeck). Sensitivity (number of MM cases with positive IR divided by total number of MM cases that underwent IHC for the marker in question) and specificity (number of carcinoma reference cases with negative IR divided by total number of carcinoma reference cases for the marker in question) were calculated for positive and negative MM IHC markers.

Survival data were obtained from the Cancer Registry of Norway. These and the microscopic data were processed in the statistical software SPSS, v. 16.0 (Producer: SPSS Inc., IBM Company, Chicago, USA), with survival being a dependent variable. Survival dependent on each single histological and IHC parameter and on a few basic clinical data was determined, and visualised by Kaplan-Meier plots. Parametric linear (Pearson) and non-parametric (Kendall's tau-b, Spearman's rho) bivariate correlations to survival were analysed with regard to significance. A multivariate linear model was calculated.

## Results

### Re-classification

**Figure 1:**
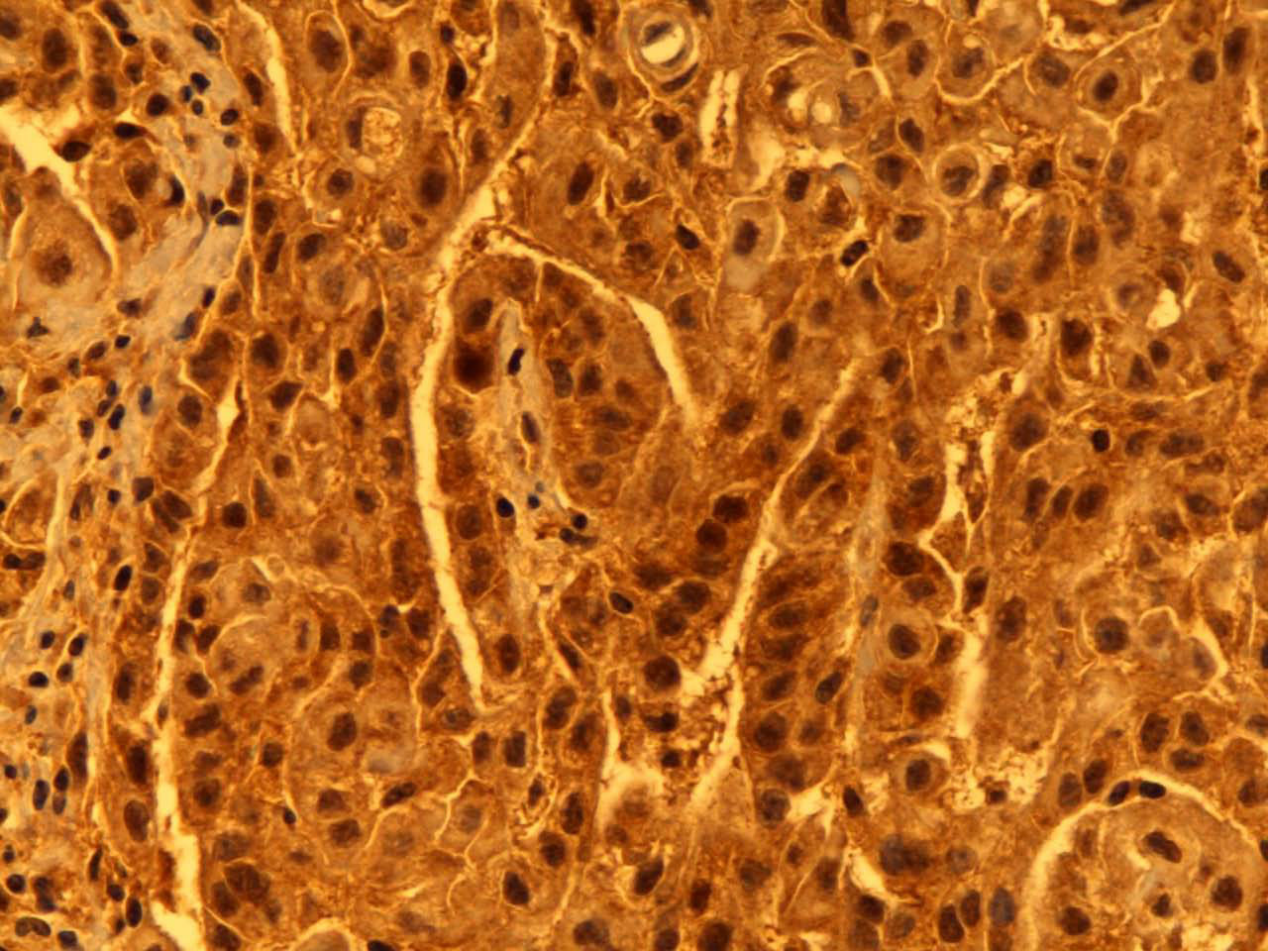
**Calretinin**. Predominantly nuclear, less cytoplasmatic staining. Epithelial type MM, pleura. Biopsy, 400×. Pat. no. 1.

**Figure 2:**
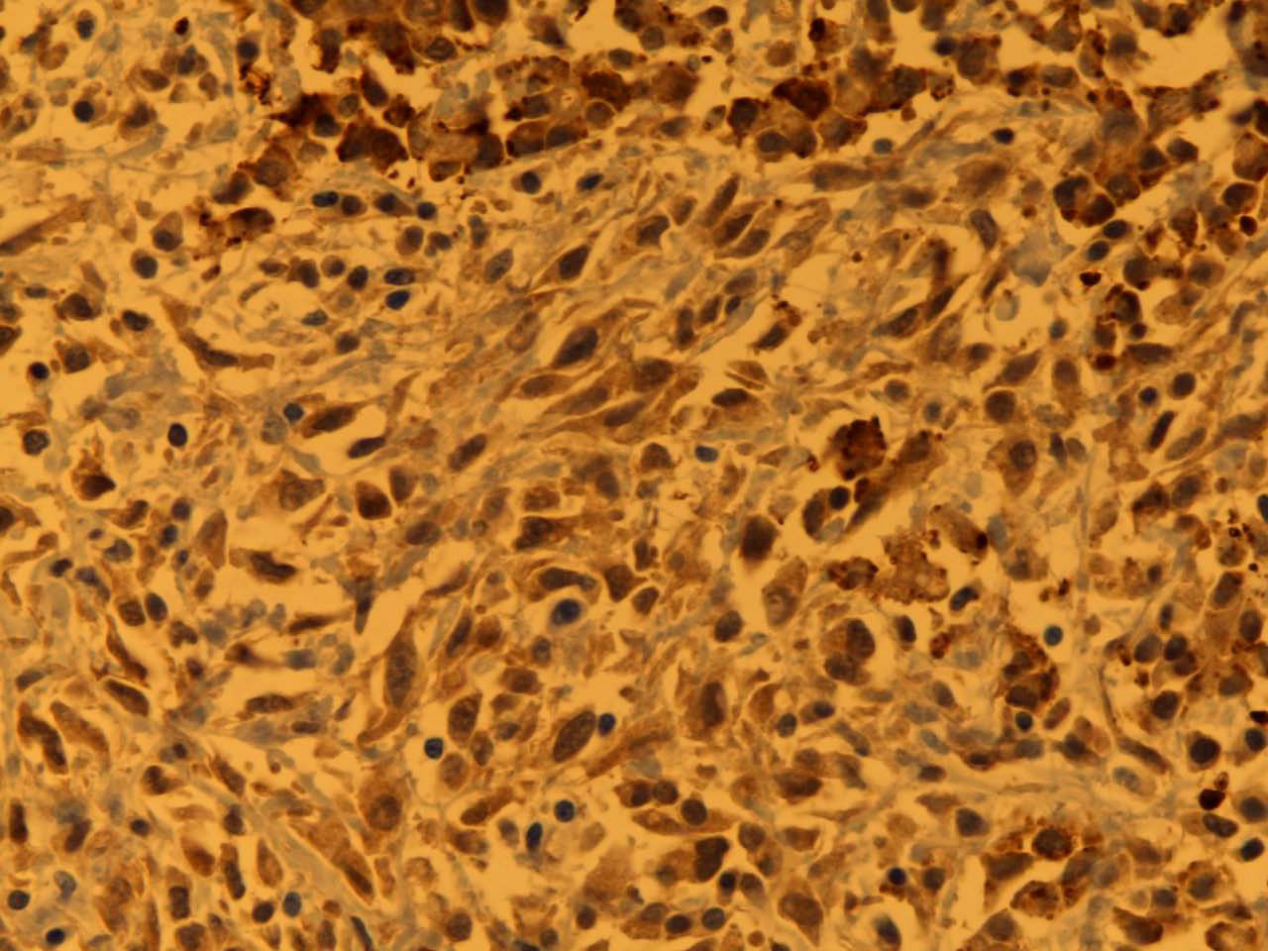
**Calretinin**. Predominantly nuclear staining. Mixed type MM, pleura. Autopsy material, 400×. Pat. no. 69.

**Figure 3:**
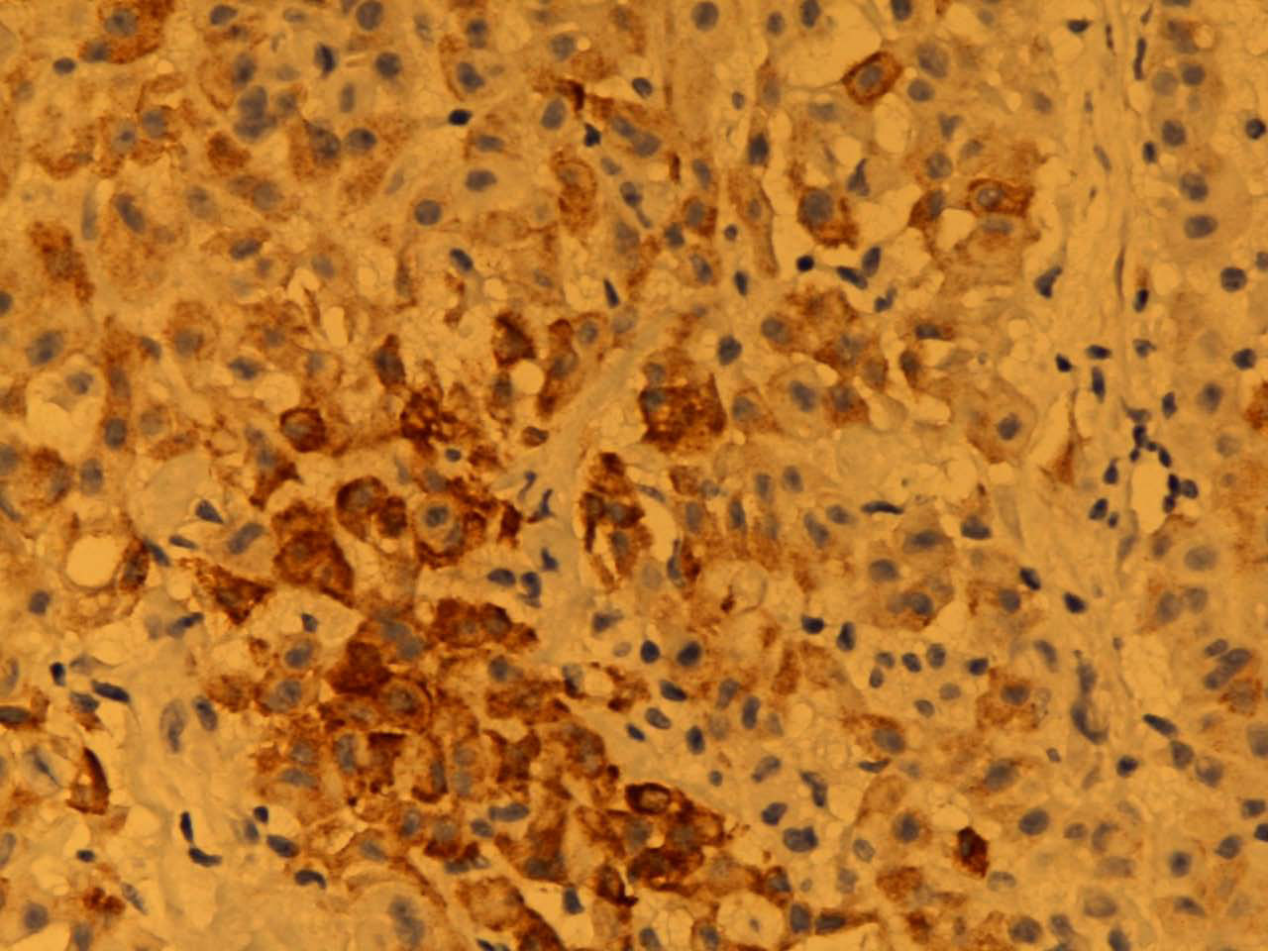
**Epithelial membrane antigen**. Various intensities of staining from negative to strongly positive, predominantly membranous. Epithelial type MM, pleura. Biopsy. 400×. Pat. no. 1.

**Figure 4:**
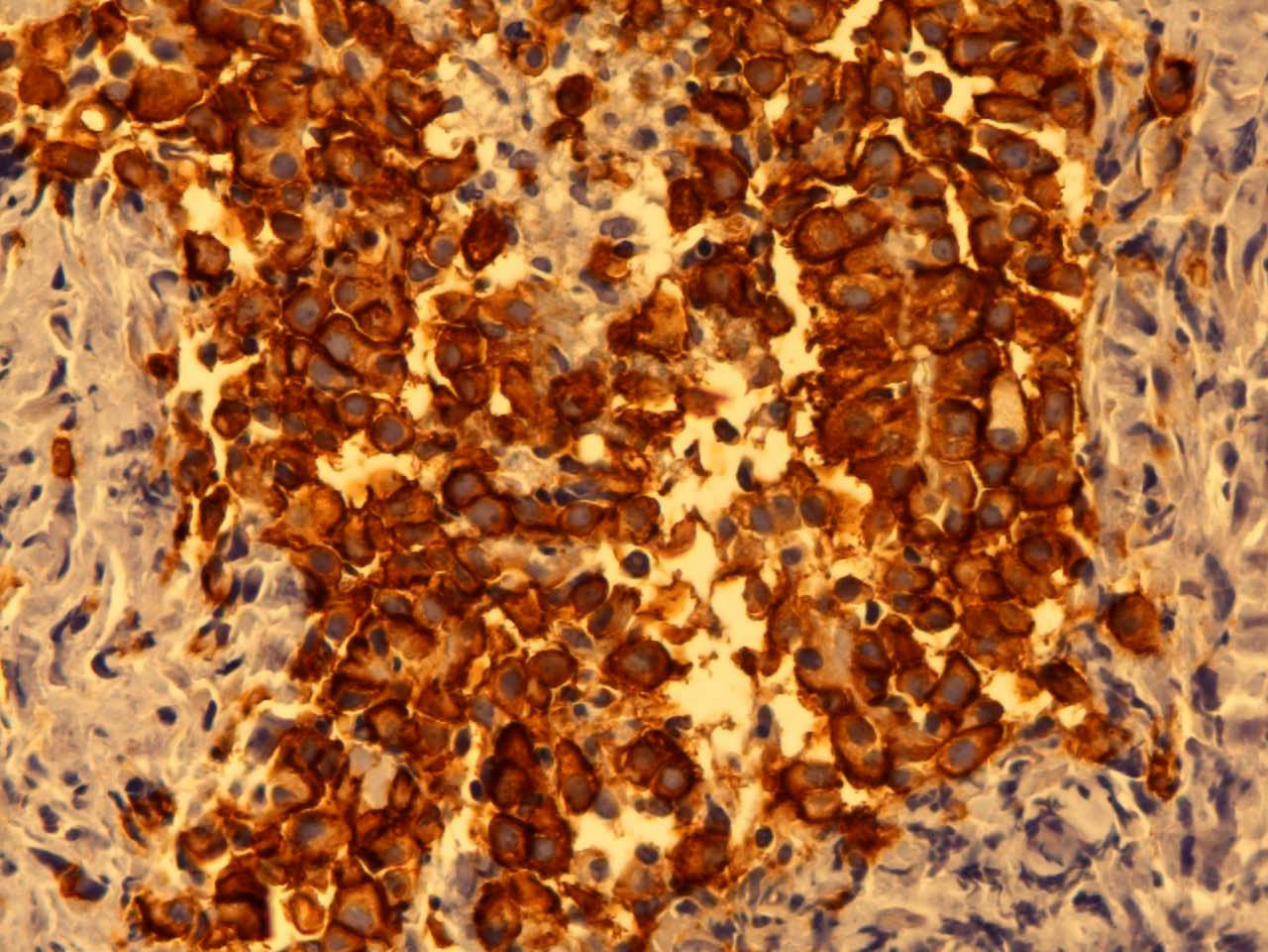
**Epithelial membrane antigen**. Partially cytoplasmatic, predominantly membranous staining. Mixed type MM, epithelial component, pleura. Biopsy, 400×. Pat. no. 33.

**Figure 5:**
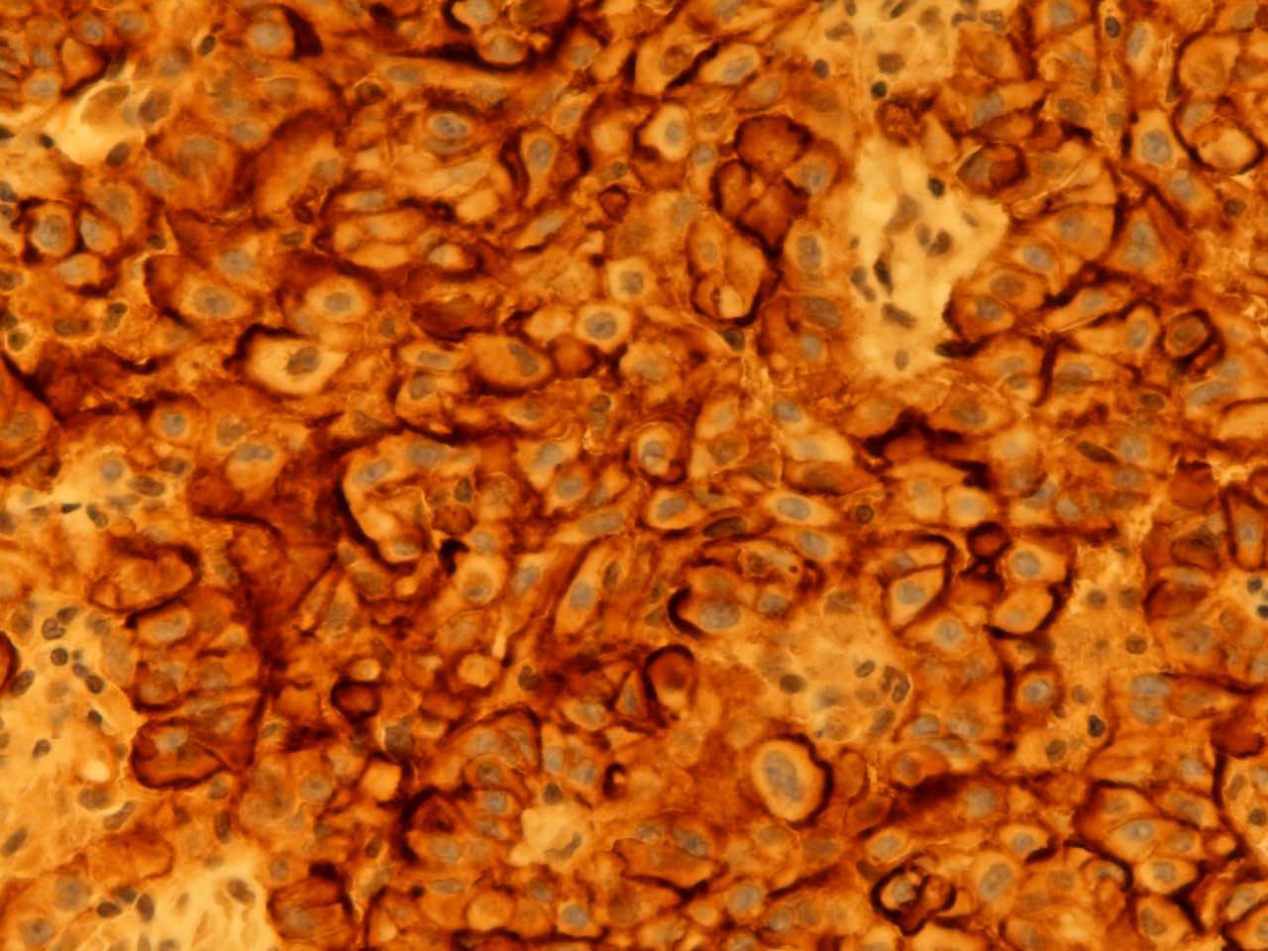
**Mesothelin**. Membranous staining. Epithelial type MM, pleura. Biopsy, 400×. Pat. no. 1.

**Figure 6:**
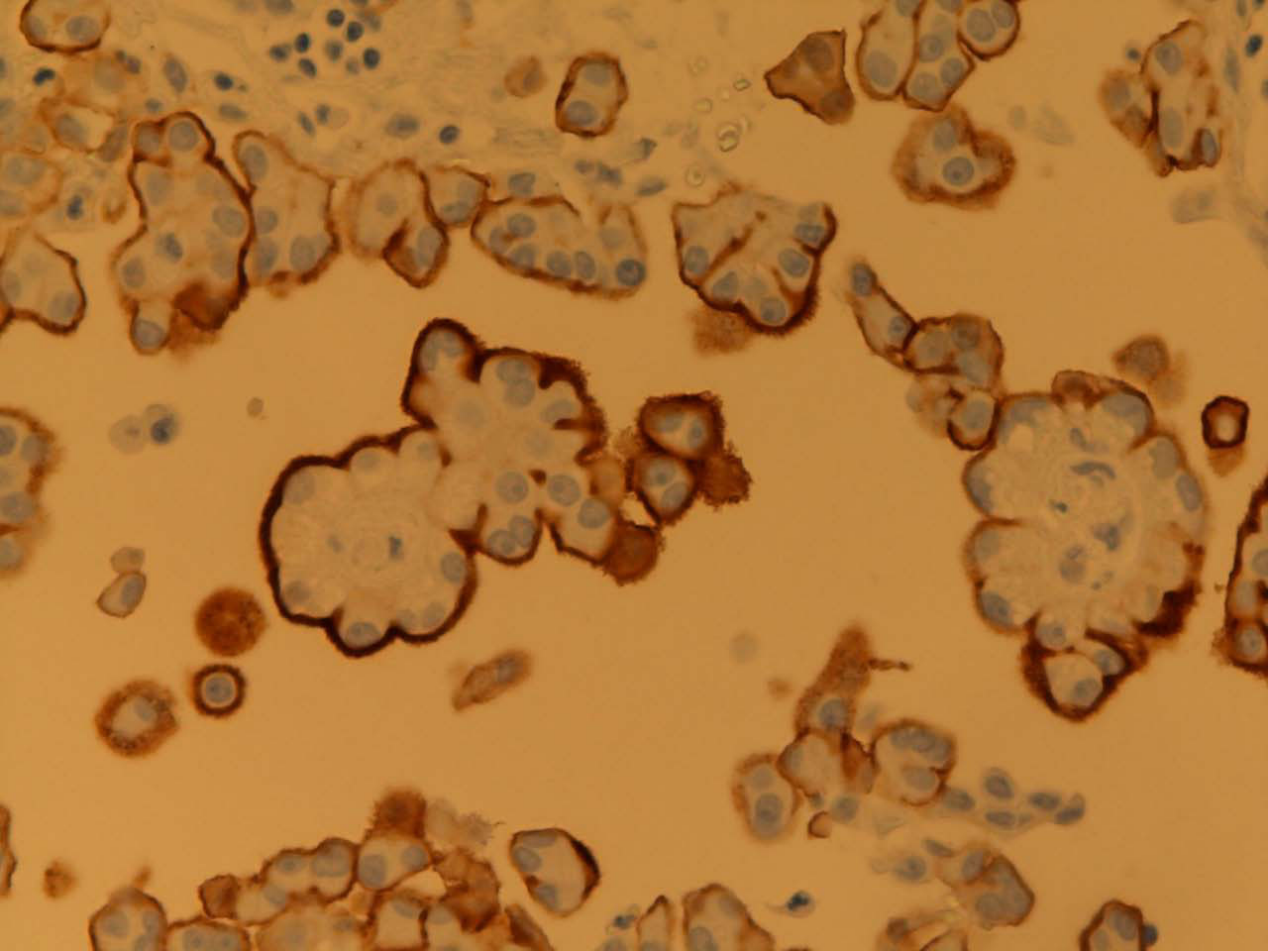
**Podoplanin**. Microvilli staining. Epithelial type MM, peritoneum. Biopsy, 400×. Pat. no. 52.

Of the 61 evaluable cases, 49 (80%; 40 men, 9 women) were diagnosed as MM, 40 (82%) as epithelial and 8 (16%) as mixed type. Of the mixed type cases, seven were localised in pleura, two of them were autopsy cases that showed secondary spreading via diaphragm into peritoneum. The remaining case was located in scrotum. All of the mixed MM types occurred in men. In one pleural case (No. 7) (2%), typing was undetermined between epithelial and mixed because of ca. 10% of sarcomatous component. All four cases of MM affection in both pleura and peritoneum were observed in men. A summary of our results is shown in Table 2 [see also Figure 1, Figure 2, Figure 3, Figure 4, Figure 5, Figure 6].

**Table 2:** Classification of cases

	Female	Male	Total
Total number of evaluable cases	11	50	61

**Validated MM**	**9**	**40**	**49 **(80%)

Epithelial/mixed/between epithelial and mixed type	9/0/0	31/8/1	40/8/1

Pleura/mediastinum/pericardium			
- Epithelial type	6/1/0	24/0/1	30/1/1
- Mixed type	0/0/0	5/0/0	5/0/0
- Between epithelial and mixed type	0/0/0	1/0/0	1/0/0

Peritoneum/pleura and peritoneum/scrotum			
- Epithelial type	2/0/-	3/2/1	5/2/1
- Mixed type	0/0/-	0/2/1	0/2/1

**Others**	**2**	**10**	**12 **(20%)

Adenocarcinoma, lung	0	2	2

Adenocarcinoma, peritoneum	1	0	1

Pleomorphic carcinoma	0	3	3

No definitive entity diagnosis, amongst them MM as one differential diagnosis	1 0	5 5	6 5

**Figure 7:**
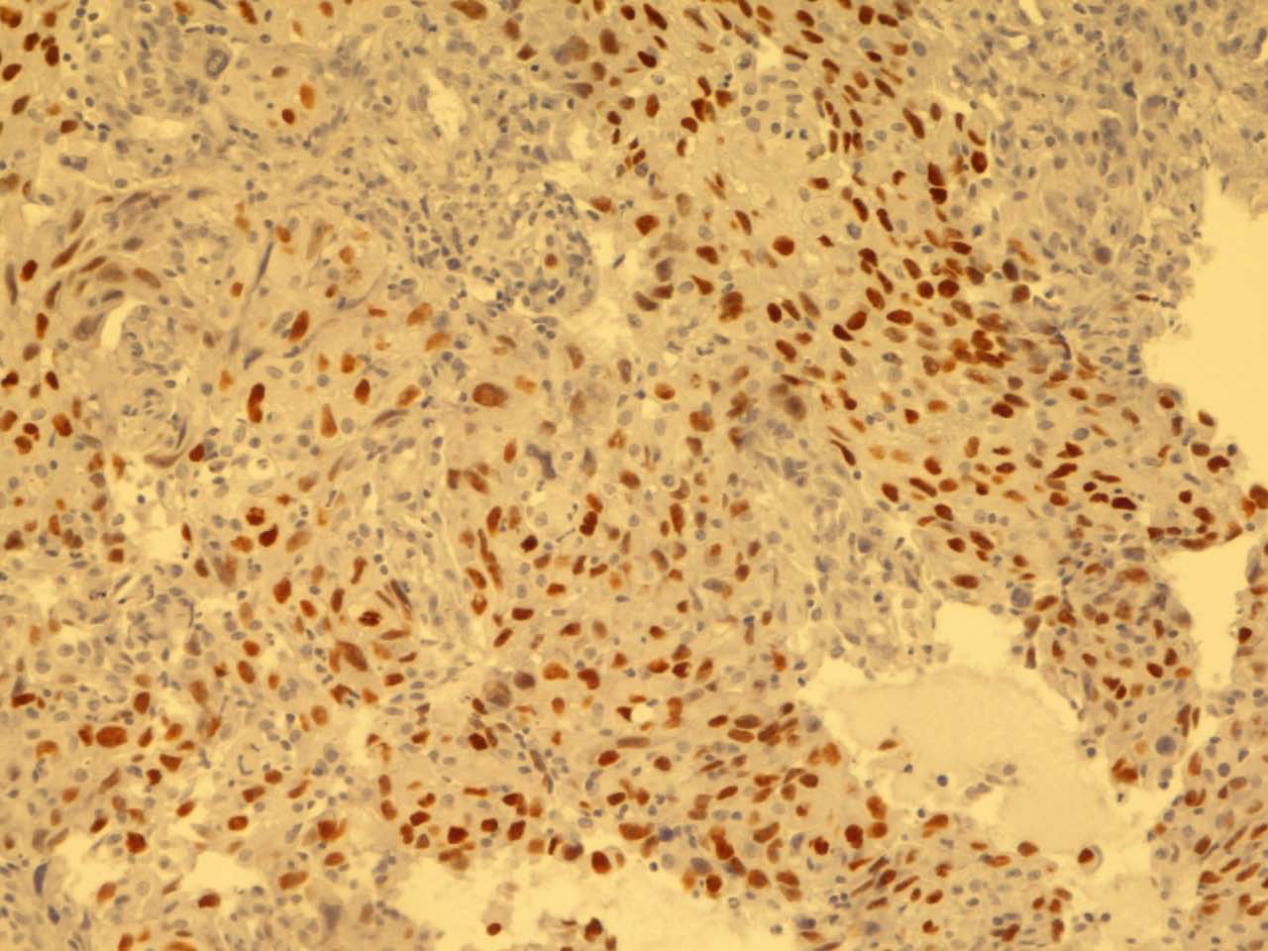
**Adenocarcinoma, lung**. TTF-1. Biopsy, 200×. Pat. no. 3.

**Figure 8:**
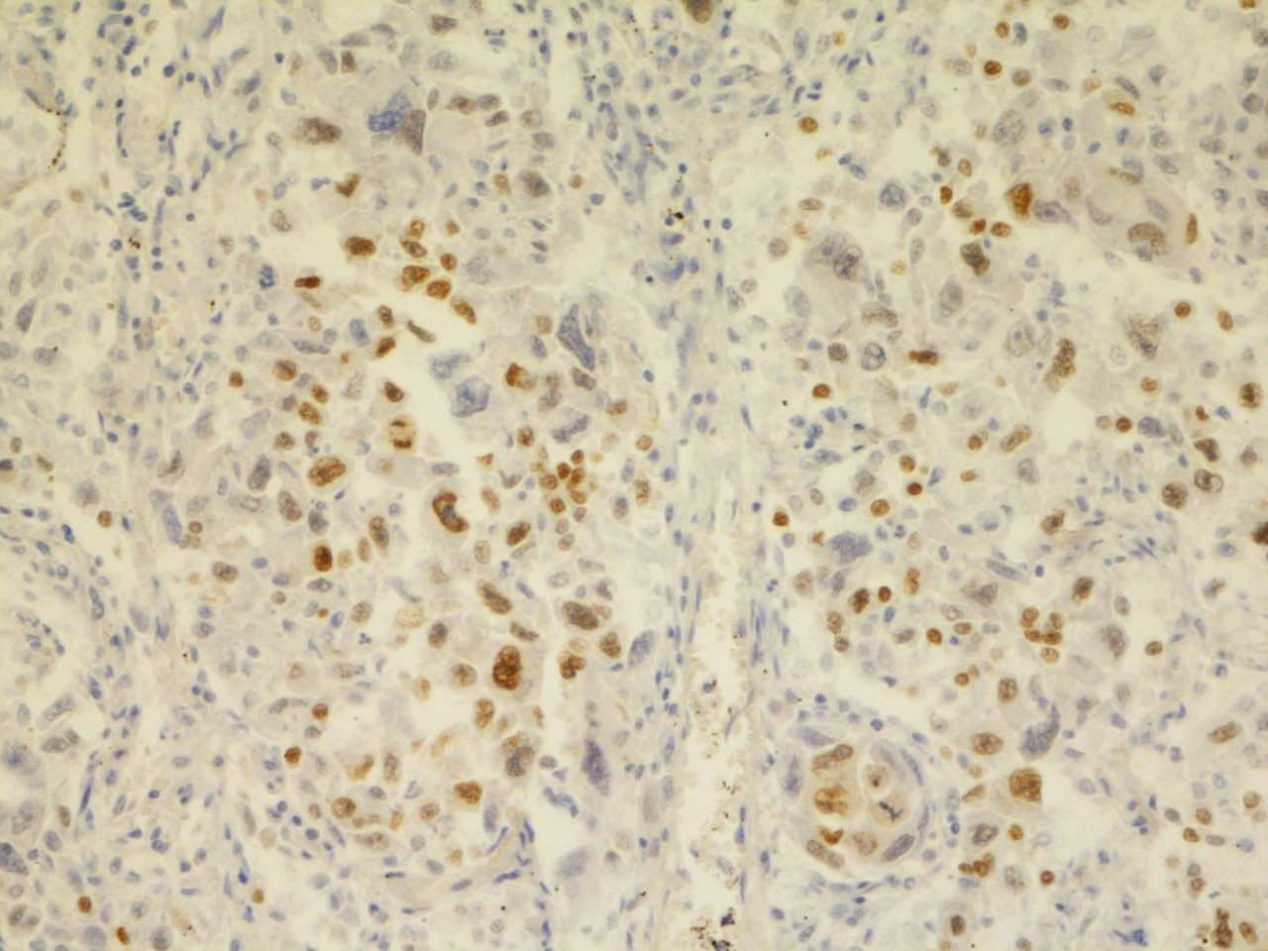
**Pleomorphic giant cell carcinoma, lung**. TTF-1. Biopsy, 200×. Pat. no. 9.

**Figure 9:**
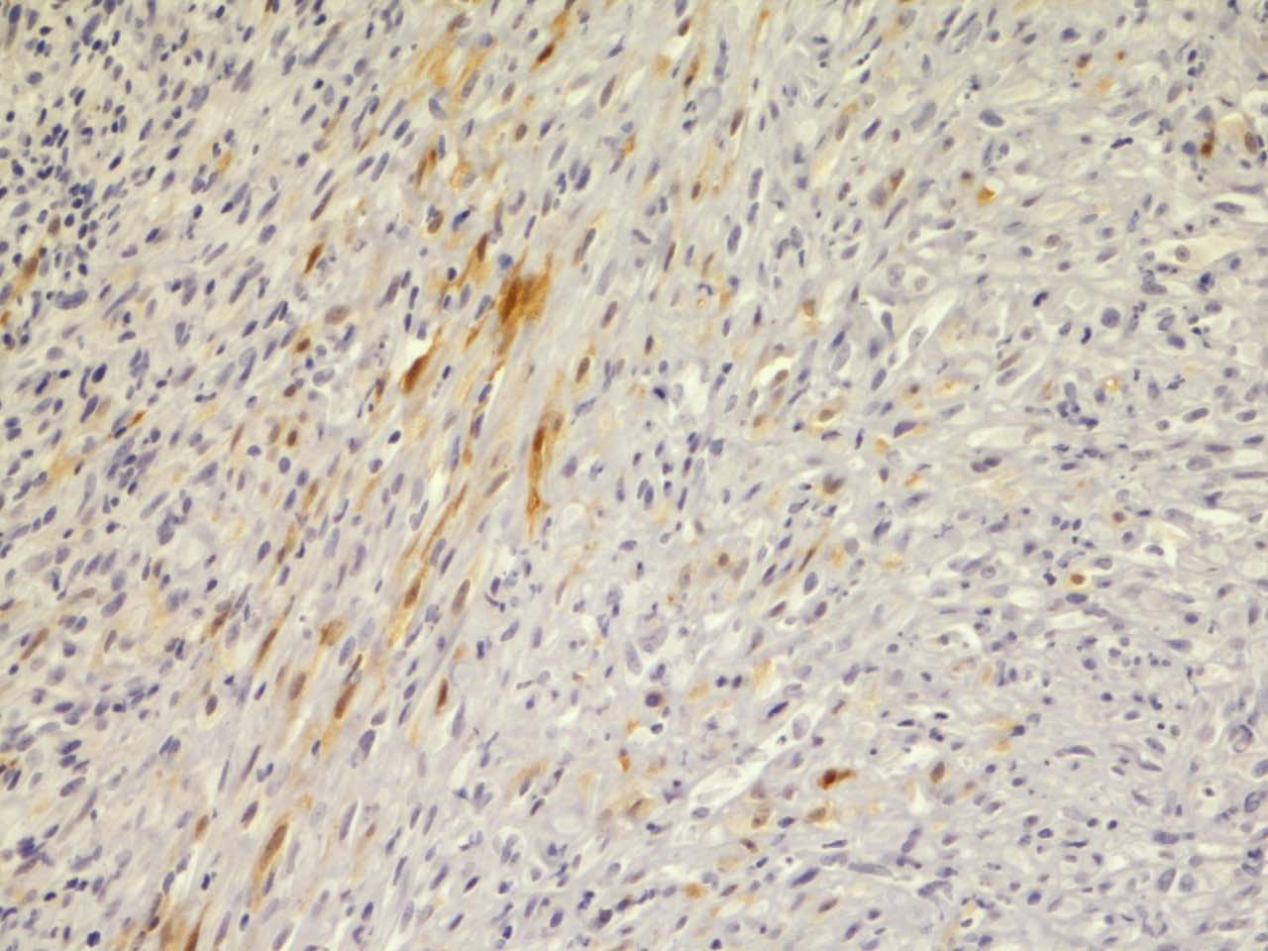
**Possible sarcomatous MM, chest wall**. Positive IR for Calretinin in some spindle cells. Biopsy, 200×. Pat. no. 57.

**Figure 10:**
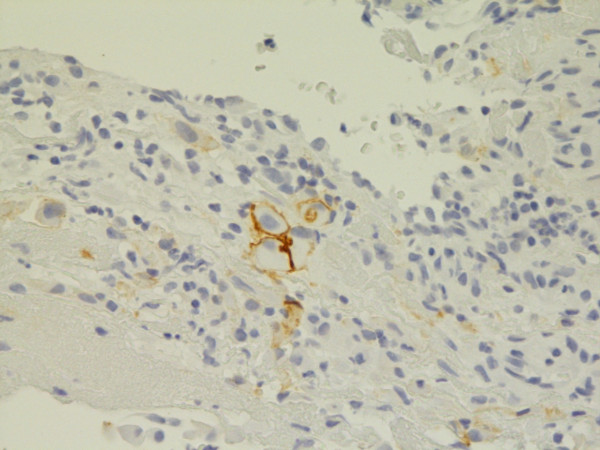
**Possible epithelial MM, lung/pleura**. Membranous positive IR for Podoplanin in some cells. Biopsy, 400×. Pat. no. 12.

**Figure 11:**
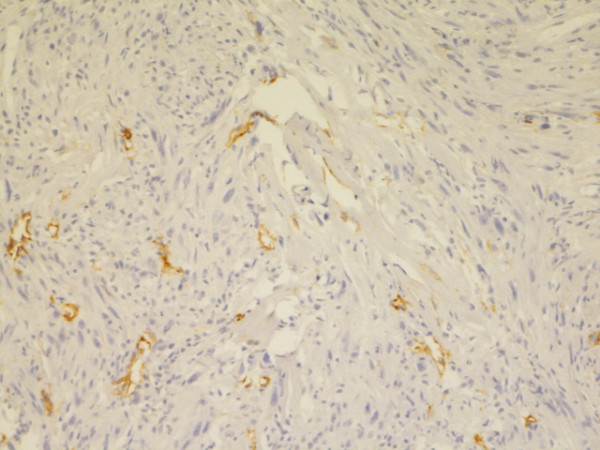
**Possible sarcoma, pleura/lung**. Positive IR for Podoplanin nearly only in lymphatic endothelium. Biopsy, 200×. Pat. no. 5.

**Figure 12:**
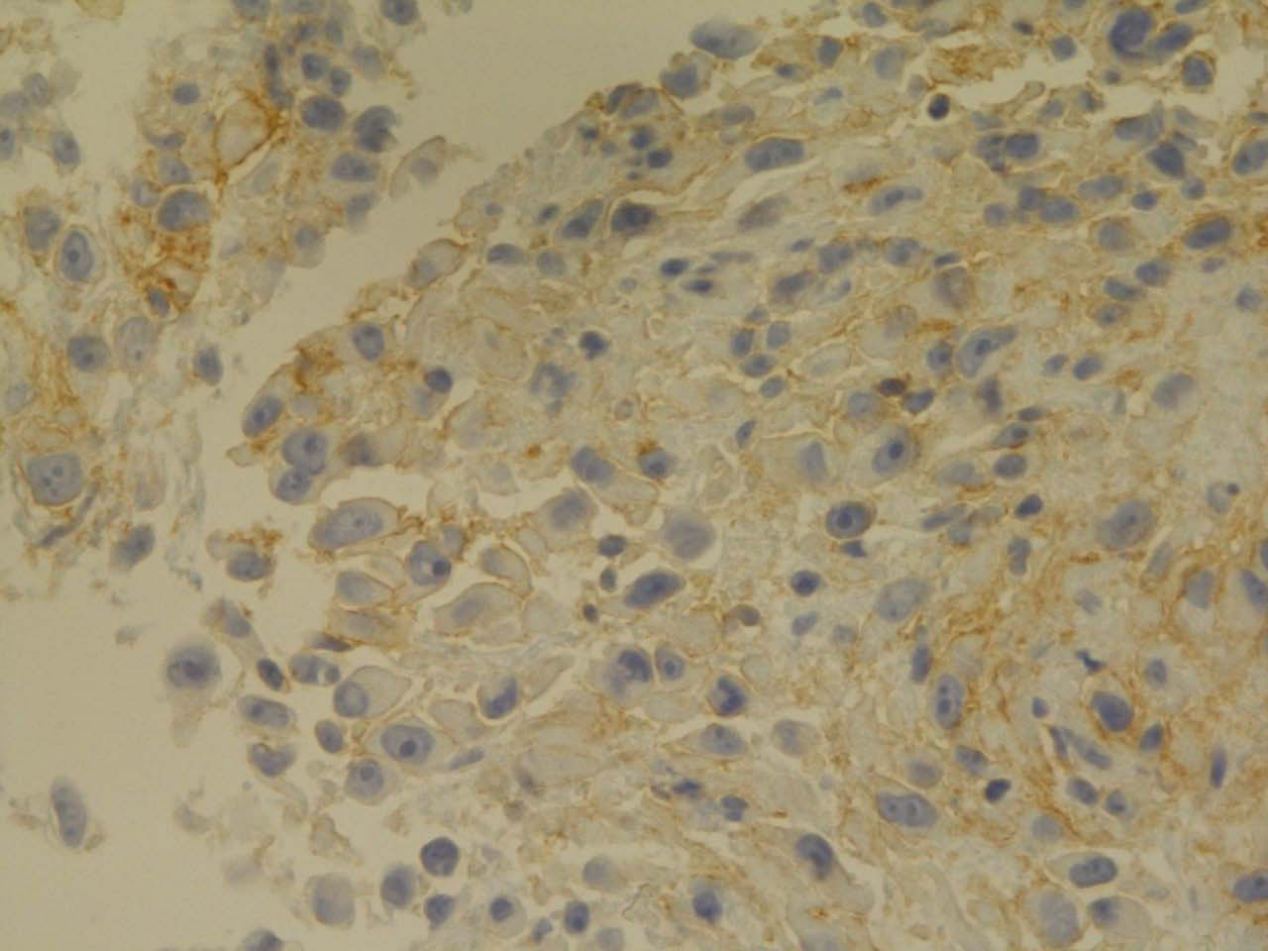
**Possible sarcoma, pleura/lung**. CD99. Biopsy, 400×. Pat. no. 32.

One earlier diagnosis of epithelial type had to be changed to mixed type when autopsy material was included (No. 11) (related to [11,24]). The remaining 12 cases (20%; 10 men, 2 women), represented various malignancies, where the primary MM diagnosis was either abolished (n = 7) or set more in doubt (n = 5). Of the five cases in which MM yet was considered possible, one was without IHC in primary diagnosis (No. 5); in the other four cases, the IHC profile was not considered being sufficient for a MM diagnosis (No. 57, 32, 63, 12). In four of these cases, sarcomatous MM and not furthermore specified sarcoma were differential diagnoses, while there were no sufficient indications for sarcomatous carcinoma (No. 5, 57, 32, 63) (Tables 3, 4) [see Figure 7, Figure 8, Figure 9, Figure 10, Figure 11 and Figure 12].

**Table 3:** Diagnostic certainty dependent on localisation of primary tumour

Localisation	Confirmed MM			Possible MM			No MM		
	Fem.	Male	Total	Fem.	Male	Total	Fem.	Male	Total
Lung/pleura	6	30	36	0	5	5	0	5	5
Mediastinum (n.s.)	1	0	1	0	0	0	0	0	0
Pericardium	0	1	1	0	0	0	0	0	0
Pleura and peritoneum	0	4	4	0	0	0	0	0	0
Peritoneum	2	3	5	0	0	0	2	0	2
Scrotum	-	2	2	-	0	0	-	0	0
Sum	9	40	49	0	5	5	2	5	7

n.s.: localisation not furthermore specified

Data are based on primarily registered MM.

**Table 4:** Possible causes of diagnostic discordance related to time of diagnosis

Possible causes	Pat. ID	Sex	Year	Our tentative HES diagnosis	Primarily registered MM diagnoses changed to:
Right histological diagnosis (without use of IHC) not accepted by clinicians, registered as MM in Cancer Registry	5 3 6	M M M	1986 1988 1988	MM/LMS MM MM/C	undetermined: sarcoma, lung or pleura/sarcomatous MM, pleura AC, lung pleomorphic carcinoma, lung

No IHC	9	M	1991	MM/C	pleomorphic carcinoma, lung

Use of non-specific markers	57 72 31 49	M M M F	1989 1996 1998 1998	MM MM/C Mal. tu. S/LMS?	undetermined: sarcoma, lung or pleura/sarcomatous MM, pleura AC, lung pleomorphic carcinoma, lung undetermined: sarcoma/sarcomatoid carcinoma, peritoneum

Differences in IR^1^	58	F	1999	MM/C	AC, peritoneum

Not appropriate conclusions^2^	32 63 12	M M M	1999 2001 2002	MM Ben./MM? Mal. Mel.	undetermined: sarcoma, lung or pleura/sarcomatous MM, pleura undetermined: sarcoma, lung or pleura/sarcomatous MM, pleura undetermined: AC, lung > MM, pleura

^1 ^between the primary laboratory and our laboratory, in addition misinterpretation of IR of another marker (one case: 58; Ber-Ep4 from negative IR in primary diagnosis to positive IR in our diagnosis, EMA positive IR only in cytoplasm)

^2 ^based on a few relevant MM markers that showed no typical MM pattern

AC - adenocarcinoma; C - carcinoma; S - sarcoma; LMS - leiomyosarcoma; Mal. - malignant; Tu. - tumor; Ben. - benign; Mel. - melanoma

None of these cases had been referred to a second opinion.

All cases were registered as MM, partially despite of histological diagnoses.

Primary histological diagnoses were:

Pat. 5: Malignant mesenchymal tumour, MM possible, no epithelial component seen

Pat. 3: Poorly differentiated carcinoma most likely, MM less likely

Pat. 6: Carcinoma most likely

Pat. 9: MM

Pat. 57: MM likely

Pat. 72: MM likely

Pat. 31: MM possible

Pat. 49: MM

Pat. 58: MM

Pat. 32: MM most likely

Pat. 63: MM, sarcomatous type

Pat. 12: MM

In one of the possible MM cases (No. 5) and two of the lung carcinoma cases (No. 3, 6) primary histological diagnoses were in accordance to our diagnoses, but had been registered as MM in the Cancer Registry. This was done apparently by clinicians who did not accept the histological diagnoses (No. 3, 6), or the likelihood consideration expressed in one of them (No. 5). Thus, in a stronger sense, only nine previous MM diagnoses (9/61, 15%), namely histological ones, were not confirmed by us. Four of them (7%) were classified as carcinomas, five (8%) as undetermined with possible MM in four of them (Table 4).

11 of the 12 mentioned, with respect to MM not confirmed cases had been diagnosed primarily in university departments of pathology. In eight of the 49 confirmed cases and in one of the not confirmed cases no university department was ever involved in primary diagnosis. Institutional affiliation of primary diagnosis is shown in Table 5.

**Table 5:** Institutions in which primary histopathological diagnostics were performed

	Institution where primary diagnosis was determined (n = 61)	
	**Non-university dept.**	**University dept.**

Original diagnostic unit	19	42

Consultation with another, university department	8	5

Re-evaluation in another, university department	2	1

Primary histological diagnosis confirmed	18 (95%)	34 (81%)

Primary histological diagnosis not confirmed	1 (5%)	8 (19%)

In all but one (No. 9, pleomorphic carcinoma of the lung) of the eight cases with diagnoses based on autopsy material, primary MM diagnoses were confirmed. In six cases the basis marker set of Calretinin, EMA, CEA and Ber-Ep4 was sufficient for diagnosis, there were five epithelial MM and one mixed MM. In one case, more IHC markers were considered being necessary, here our diagnosis was mixed type MM.

### Sensitivity and specificity of IHC markers

Based on the five pulmonary carcinoma cases, specificity was lower than sensitivity for Podoplanin, EMA and monoclonal CEA, but 100% for Calretinin and Mesothelin. Concerning sensitivity, the most reliable single markers amongst the six markers were Calretinin and CEA. However, in two of 49 MM cases Calretinin showed positive IR only in a few tumour cells (Table 6). No difference in MM IR for monoclonal and polyclonal CEA was detected, although inflammatory cells gave a positive IR for polyclonal, and not for monoclonal CEA.

**Table 6:** Sensitivity and specificity, confirmed MM cases

Marker	Sensitivity, MM			Specificity, against carcinoma		
**Positive MM markers**	**positive IR, number**	**positive IR, %**	**literature data, %**	**negative IR, number %**	**negative IR, %**	**literature data, %**

Calretinin, nucleus	49/49	100	82^1^ 96.4^2^	5/5	100	85^1;2^

EMA, cell membrane	43/49	87.8	84^3^	3/5	60	56^3^

Podoplanin, cell membrane	44/46	95.6	86^4^	2/3	66.7	100^4^

Mesothelin, cell membrane	44/48	91.7	100^5^	4/4	100	61^5^

Thrombomodulin, cell membrane	7/7	100	61^1^	2/5	40	80^1^

HBME-1, cell membrane or cytoplasm	(4+2)/8	75	85^1^	(2-1)/5	20	43^1^

CK5/6	3/3	100	83^1^	3/5	60	85^1^

**Negative MM markers**	**negative IR, number**	**negative IR, %**		**positive IR, number**	**positive IR, %**	

CEA, monoclonal	49/49	100	81^1^	1/5	20	97^1^

Ber-Ep4	40/49	81.6	80^1^	3/5	60	90^1^

CD15	7/7	100	84.9^6^	1/5	20	81^6^

Sialosyl-TN	6/7	85.7	76.5^6^	1/5	20	81^6^

TTF-1	3/3	100	72^1^	5/5	100	100^1^

Specificity, own results: against AC and pulmonary pleomorphic carcinoma.

Comparison to some selected data from literature.

^1 ^King et al. 2006, meta-analysis of literature data, MM with epithelioid areas against pulmonary AC [27].

^2 ^Takeshima et al. 2009: sensitivity epithelial/mixed MM 96.4%/90% [1]. Yaziji et al. 2006: sensitivity 95%, specificity against AC 87%, both with 10% positive cutoff [28].

^1, 2 ^Calretinin IR not specified.

^3 ^Wick et al. 1990, related to epithelial MM and pulmonary AC [29].

^4 ^Ordóñez 2005, related to epithelial MM and AC [21].

^5 ^Ordóñez 2003a, related to epithelial MM and pulmonary AC [30].

^6 ^Brockstedt et al. 2000, related to epithelial/mixed MM and AC [31].

### Marker expression in relation to grade of atypia

Two of 49 MM cases (4%) showed the lowest degree of cytological atypia in the material, grade 1 and 1.5, and three of them (6%) high atypia, grade 3. The expression of Podoplanin and Mesothelin in the grade 3 cases was in all cases low compared to the grade 1 till 2 cases.

In five cases there were a few MM cells, near 0%, with positive IR for Ber-Ep4, in four cases some more, but less than 25% of MM cells. IR for CEA was negative in all cases (not shown). Of the four positive markers, Calretinin and EMA were most expressed even in poorly differentiated MM (Table 7).

**Table 7:** Histological grading of atypia and immune reaction for main MM markers

Grade of atypia (n = 49)	Cases (n)	Percent (%)	Calretinin, nucleus (n = 49)	Epithelial membrane antigen, cell membrane (n = 49)	Podoplanin (n = 47)	Mesothelin (n = 48)	Ber-Ep4 (n = 49)
1	0	0	-	-	-	-	-

1.5	2	4	4 4	1 3	4 3	4 4	0 0

2	25	51	2.84 (1...4)	2.00 (0...4)	2.65 (1...4)	3.46 (0...4)	.08 (0...1)

2.5	19	39	2.26 (near 0...4)	2.16 (near 0...4)	1.74 (0...4)	3.11 (0...4)	.11 (0...1)

3	3	6	2 3 1; 1	2 2 2; 1	1 1 1	2 1 1	0 0 0

Number of MM cells with positive IR, semiquantitatively on a 0...4 scale (steps of 25%). For grades 2 and 2.5, average values and overall minimum-maximum ranges are indicated. IR for CEA was negative in all of the cases.

No obvious differences in IHC staining intensity and distribution were seen between biopsy and autopsy material, even if there were prominent signs of autolysis in the latter one, or related to the age of the tissue material (not shown). However, there were only four cases with both biopsy and autopsy material, with partially insufficient amount of the former one. A systematic comparison of marker expression in these cases as in [32] was not performed.

### Use of IHC in primary diagnosis in the confirmed MM cases

In at least 26 cases (53%) between one and 18 IHC markers had been applied (Table 8). In all but one of the confirmed cases, the positive marker combination Calretinin and EMA could have been replaced by the combination Podoplanin and Mesothelin, leading to the same diagnosis. In one case (autopsy, No. 70) which showed only a few nuclei with positive IR for Calretinin and positive membranous IR for EMA in less than 25% of cells, the combination Podoplanin and Mesothelin, with IR in two and three quartiles of tumour cells, respectively, would have been a better option. However, in a case of MM of tunica vaginalis testis (biopsy, No. 25) with only a few cells showing the same kind of IR for Calretinin and EMA, IR for Podoplanin and Mesothelin were completely negative.

**Table 8:** Application of IHC in primary diagnosis in confirmed cases (some patterns, partially overlapping cases)

Year	Confirmed cases	IHC/markers in primary diagnostics
1980-2002	49 (100%)	
	19 (39%)	No IHC performed
	4 (8%)	No information available about eventual IHC use
	26 (53%)	IHC, 1 till 18 markers
		***No specific positive MM markers***
1988-1999	7 (14%)	CEA, Vimentin and Pan-Cytokeratin
		***Broad spectrum of non-specific markers***
1996, 1997	2 (4%)	15/18 markers, 1^st ^one incl. positive marker HBME-1, otherwise no specific positive MM markers
		***Positive MM markers***
1998-2002	8 (16%)	Calretinin, in all but one (1998) of cases combined with other markers, two cases with another positive marker - Thrombomodulin and HBME-1, respectively (n = 1-7)
		***Negative MM markers***
1988-2002	17 (35%)	CEA, in all but one (1990) of cases combined with other markers (n = 1-18)
1996, 2002	2 (4%)	CD15, combined with, amongst other markers, CEA and Ber-Ep4 (n = 15, 7)
1996-2002	10 (20%)	Ber-Ep4, combined with other markers (n = 4-15)

### Use of IHC in primary diagnosis in the not confirmed MM cases

Four of the 12 not confirmed cases from between 1986 and 1991 lacked IHC. In four cases from between 1989 and 1998, non-specific IHC markers were applied. One case from 1999 (No. 58) showed different IR for one crucial negative MM marker, Ber-Ep4, which was described as negative in primary diagnosis, but which showed positive IR in our IHC staining. The positive marker EMA showed positive IR, but only in cytoplasm. In the remaining three cases from between 1999 and 2002 which all are of undetermined entity, combinations of relevant IHC markers including Calretinin, EMA and Ber-Ep4 were used, but Calretinin showed negative IR and EMA positive IR in cytoplasm. In five of the six undetermined cases, MM is still one of the differential diagnoses (Tables 2, 4) [see Additional file 1].

### Tentative diagnosis without IHC

In 46 of the 49 cases confirmed by us (94%) we previously also had diagnosed MM as most likely based on HES slides only. In the remaining three cases, MM was a differential diagnosis together with carcinoma. In eight of the 12 not confirmed cases (67%), MM was one of our tentative differential diagnoses. In three of these cases (25%), MM was our only tentative diagnosis. Two of the latter cases remained undetermined, one case showed most likely pulmonary AC after IHC (Table 4).

### Survival

Overall median survival was 12.5 months, with almost no difference between men and women (12.2 and 12.5 months, respectively). Pleural MM survival ranged from 0 to 44 months, with a median of 12.5 months. Mediastinal, pericardial and scrotal MM (1/1/2 cases) survival was 2.5, 4, 6 and 10 months, respectively. The longest median survival, 51.8 months, was observed in peritoneal MM, with a minimum of 5.5 months in men and 31.5 months in women.

Survival was in the statistic median and mean poorer when IHC Mesothelin expression was reduced, this was also true for Calretinin in nuclei and even more for Podoplanin, which was the only IHC marker showing significant bivariate correlation [11]. Mixed type, presence of spindle cells, high cellular atypia, more than one mitosis per 10 HPF, presence of atypical mitoses, necrosis, presence of regional lymph node and/or distant metastases and elevated age at diagnosis were correlated with poorer survival. Peritoneal MM was correlated with longer survival than MM in all other localisations. All of these parameters, except the number of atypical mitoses, showed significant bivariate correlation with survival, too (Table 9).

**Table 9:** Survival-related single factors, tendencies of correlation

Factor	Relation to survival, clear tendency	Significant (only) in bivariate correlation with survival (two-tailed), level
**IHC markers, semiquantitatively**, when increasing number of cells with positive IR:		

Calretinin, nucleus	↑	-

Calretinin, cytoplasm	↔	-

EMA, cell membrane	↔	-

EMA, cytoplasm	↔	-

Podoplanin, cell membrane	↑	0.001

Mesothelin, cell membrane	if 0-50%: ↓ 51-100%: ↑	-

Ber-Ep4, general	if 1-25%: (↓)	-

**Histological type**		

Epithelial	↑	0.05 (non-parametric)

Mixed	↓	0.01 (non-parametric)

**Histological markers**		

a) when increasing:		

Degree of cellular atypia	↓	0.001

Spindle cells, semiquantitatively	if 0: ↑; otherwise: ↓	0.001 (non-parametric)

Number of mitoses (10 HPF)	if 0 or 1: ↑; otherwise:↓	0.05 (Spearman's rho)

Number of atypical mitoses (10 HPF)	if 0: ↑; otherwise: ↓	-

Necrosis	↓	0.01

Mucus, cytoplasm, number of cells	if high: (↓)	-

Mucus, extracellular, number of cells	↔	-

Accompanying inflammation, intensity	↔	-

Accompanying inflammation, focality	↔	-

b) if present:		

MM spreading, perineural	(↔)	-

MM spreading, intralymphangic	(↔)	-

MM spreading, intralymphangic and venoles	(↓)	-

No/no evident MM spreading	↑	-

Accompanying inflammation, active	(↔)	-

Accompanying inflammation, non-active	↔	-

**Localisation of primary tumour**		

Pleura	↓	-

Peritoneum	↑	0.01 (parametric) 0.05 (non-parametric)

Pleura and peritoneum	(↓)	-

Mediastinum, not specified	(↓)	-

Pericardium	(↓)	-

Scrotum	(↓)	-

**Metastasis**		0.05 (non-parametric)

yes	↓	

no/not known	↑	

**Sex**		-

Female	↔	-

Male	↔	-

**Age at time of diagnosis, when increasing:**	↓	0.01

In parentheses: very few cases

Survival is given per April 21, 2008.

Multivariate linear analysis revealed a model to which only localisation in peritoneum, cellular atypia and necrosis contributed significantly, namely at <0.05 levels (at 0.001 level, 0.031 and 0.032, respectively), not the other parameters that had shown significant bivariate correlations with survival (0.2 till 0.9 levels in multivariate analysis).

Women were only represented in the primary diagnosis age groups of 45 till 59 years, men in all age groups from 45 till 79 years. There was a tendency of longer survival in men then in women in the age groups of 45-54 years. No relevant differences in survival could be observed between men and women, when results were stratified according to localisation in pleura or peritoneum. All four cases of MM affection in both pleura and peritoneum were observed in men. Concerning epithelial type MM, no survival difference was seen between men and women, mixed type MM was only observed in men.

Survival in lung AC was 1.5 and 7.5 months, in pleomorphic lung carcinoma 1; 3.5 and 13 months, in possible peritoneal sarcoma/sarcomatoid carcinoma 2 months, in possible pulmonary/pleural sarcoma/sarcomatous MM (No. 5, 57, 32) 3, 1 and 1 months, in possible pulmonary AC/pleural MM 2 months and in peritoneal AC at least 100 months (related to Table 4).

## Discussion

In this study, we re-evaluated a Norwegian tissue material with an earlier diagnosis of MM which was regarded as potentially suitable to further molecular studies aiming at a deeper understanding of MM biology [4,11,24].

All patients were donors to the Norwegian Janus serum bank, as the only applied selection criterion of the MM material [33].

Our material is not representative concerning MM type distribution, as there have been diagnosed more mixed and sarcomatous types in larger newer studies (Roberts et al. 2001: 73% epithelial/11% mixed/10% sarcomatous/6% desmoplastic; Borasio et al. 2008: 67% epithelial/23% mixed/10% sarcomatous), so far as one supposes that similar diagnostic criteria were applied, which is not necessarily the case [32,9]. However, when including the four cases of possible sarcomatous type in our study, proportions would be more similar (n = 40/8/4; 77%/15%/8%). MM organ distribution seems to reflect the overall incidence [12].

Due to scarce material, some relevant antibodies as WT-1, h-Caldesmon, and others, as well as specific histochemical reactions on glycoproteins and hyaluronic acid could not be applied, despite of their diagnostic value [34-36]. More extensive diagnostics including genetic testing, fluorescence in situ hybridisation (FISH) and electron microscopy (EM) in order to possibly reach a final entity diagnosis in the unclear cases, were not carried out, since this was not a concern in this study where the main point was to exclude all insecure and non-MM cases by a high degree of likelihood [37,23,39].

Our choice of Calretinin, EMA, CEA and Ber-Ep4 was determined by their diagnostic value, as recently established by Brockstedt et al. who singled out CEA and Calretinin as the most informative markers in differential diagnosis between MM and AC [31]. Their next most valuable markers were CD15 and EMA, while Ber-Ep4 was fifth. The reason to choose Ber-Ep4 instead of CD15 as one basic negative marker was to test whether Ber-Ep4 really could replace CD15 in this constellation, as it according to our knowledge apparently has been used much more. However, direct comparison was only possible in seven cases where also IHC on markers no. 5-8 (HBME-1, Thrombomodulin, CD15 and Sialosyl-TN) was performed. As there was partial positive IR for Ber-Ep4 in two verified MM cases, but negative IR for CD15 in all of the seven verified cases, we too experienced that CD15 was a more reliable marker than Ber-Ep4. In four of the seven cases (no. 11, 25, 39, 62), CK5/6 and TTF-1 were considered necessary for confirming the diagnosis. Mesothelin was chosen in order to compare IR on MM tissue with serum levels and to evaluate it as an IHC marker, Podoplanin for the latter reason [4,11,30,40,20,21].

WHO/IMP demand a minimum of 10% sarcomatous cells to define a mixed MM. In order to reduce MM typing problems, it has been proposed that at least 30-40% of a sarcomatous component should be present to call it a mixed type [41]. This would also be appropriate in order to avoid taking sarcoma-like stromal reactions as true sarcomatous MM components. However, in small biopsies, even such a component ratio may happen to be not representative at all. Which cytological and histological patterns are considered to represent a sarcomatous MM component or type is also varying [26,42]. Such variations in opinions, approaches and definitions may cause significant differences in the MM type frequencies making it difficult or impossible to compare different studies.

We detected a 15% discrepancy of former histological MM diagnosis. This could be explained by either 1) lack of IHC (n = 1), 2) use of non-specific IHC markers (n = 4), 3) differences in IR (n = 1) or 4) not appropriate conclusions based on appropriate markers (n = 3) influencing the primary diagnosis.

The case of the first group was from the period before 1998 when no specific IHC markers were available for actual differential diagnostics in routine pathology. Today it would be quite uncommon not to use IHC at all in MM diagnostics, at least in industrialized Western countries. At the same time, this illustrates the problems that may occur in countries with a less developed health care system where IHC might not be affordable.

As to the non-specific markers used in the second group that covers a period until 1998, this was due to the lack of more specific markers.

Differences in IR as in the third group and not appropriate conclusions as in the fourth group may occur also today. The former may be reduced by the use of standardized equipment and workflow in IHC laboratories, e.g., by IHC staining machines.

In two cases where there had been used non-specific markers in primary IHC (No. 31, 72), the later (1999) introduced marker TTF-1 was crucial to establish the pulmonary carcinoma diagnosis. This also happened in three of the cases that had lacked IHC (No. 3, 6, 9) (table 4) [43].

Sarcoma, not further specified, was a differential diagnosis in five of the six cases with undetermined re-evaluation diagnosis, together with sarcomatous MM of pleura/lung in four of them. This reflects the potential diagnostic difficulty in a part of the sarcomatous lesions of pleura and lung even when IHC is available. In the one CK-negative (no. 32) and in the four CK-positive (no. 5, 49, 57, 63) spindle cell tumour cases, Comparative Genomic Hybridisation (CGH) might have allowed distinguishing between sarcomatous MM and other entities [37].

The high percentage of cases in which university departments were involved amongst the cases not confirmed by us (8/9, 89%, with respect to histology) may be partially due to the generally high rate of MM diagnoses in which a university department was involved (52/61, 85%). Amongst these cases, there were no consultation cases sent from non-university departments. Apparently, difficult cases were sent from non-university departments to university departments for a second opinion, or primary diagnosis. However, in none of the not confirmed cases, a second opinion was asked for.

Whereas there does not seem to exist poorly differentiated epithelial type MM without any expression of one or more of such positive markers, as, e.g., Calretinin, WT-1, Mesothelin or Podoplanin, the complete absence of IR for such markers is allowed for the sarcomatous type by the above mentioned definition, as far as there is a positive IR for broad-spectrum cytokeratins. This would indeed mean that, with increasing dedifferentiation, specific mesothelial markers disappear, while cytokeratins are preserved even in poorly differentiated sarcomatous MM. However, in cases with completely negative IR for mesothelial markers, there would not be any sufficient histological evidence for the presence of MM. The clinical and radiological picture would then play a major role in tentative diagnosis.

In a study by Blobel et al., all forms of MM expressed CK 8 and 18, and most of them CK 7 and 19, the same CK profile as in AC [44]. In addition, CK 5 was expressed in all epithelial and most of the mixed types. CK 4, 6, 14 and 17 were also present in variable amounts (see also [45]). The authors concluded that MM has to be defined as a variant of carcinoma. Histologically, this is indeed a reasonable conclusion even from today's point of view [46]. Sarcomatous MM could then be regarded as mesothelial sarcomatoid carcinoma. On the other hand, several sarcomas also express cytokeratins.

In our material, 19 MM diagnoses (39%) from the whole period (1980-2002) which were established without any use of IHC could be confirmed by us. All of them were also tentatively diagnosed by us as most likely MM in HES only slides. Apparently, the microscopic pictures in these cases were sufficient in order to recognize epithelial or mixed type MM. Related to our final diagnoses, in about 94% of the cases of epithelial MM, our preliminary, tentative HES-based entity diagnoses were correct. There would remain about 6% of diagnostic failure regarding the epithelial subtype alone. Most often this would concern the differential diagnosis between MM and AC, which may have therapeutic implications. In the differential diagnosis of sarcomatoid lesions, without IHC, there would be a much higher error rate when the pathological diagnosis is based only on HE or HES stained slides. This underlines the value of IHC in MM diagnosis, especially if there is any doubt concerning the HES picture.

Before more specific MM IHC markers were introduced, in the confirmed MM cases, marker combinations consisting of CEA and Pan-cytokeratin or Vimentin were applied. Here, negative IR on CEA and the morphological picture seem to have been crucial for the MM diagnosis, since there may be positive IR of both Pan-cytokeratin and Vimentin in carcinomas and sarcomas. Specific positive MM markers were not available until approximately 1998 when Calretinin was introduced in Norway.

All of our confirmed cases showed at least focally positive IR for Calretinin. However, there were no cases amongst the not confirmed cases in which missing Calretinin IR would have been the only exclusion criterion [see Additional file 1].

A basic IHC marker combination of two positive (e.g., Calretinin, EMA membranous) and two negative (Ber-Ep4, CEA) markers was well applicable to distinguish most of epithelial MM from other differential diagnostic possibilities. When it is necessary to distinguish MM from pulmonary AC, and when a synovial sarcoma can be ruled out, positive IR for TTF-1 would be useful for a pulmonary carcinoma diagnosis. With the exception of EMA, all of the mentioned markers are among the mesothelial and epithelial markers focused on by the WHO panel [3]. Meanwhile, positive membranous IR for EMA scored high as a positive MM, or mesothelial marker in the study of Brockstedt et al. that is based on a large MM material, and it is also mentioned as usually negative in benign mesothelial proliferations in the recent IMIG recommendations [31,23]. EMA is also a recommended marker in the recent ERS/ESTS guidelines [15].

Based on our results, only Calretinin and CEA showed 100% sensitivity (Table 6). In two cases, only a few MM cells showed positive nuclear IR for Calretinin. One of them (no. 25) was a mixed MM of tunica vaginalis testis. In a study of Winstanley et al. on 20 tunica vaginalis testis cases, all of them showed negative IR for CEA and positive IR for Calretinin, but only 16 (80%) nuclear IR for Calretinin [47]. Our second case (no. 75) was an epithelial MM of pleura that also showed only a few cells with positive membranous IR for EMA.

Because of the predominating purpose of re-evaluation, the used tissue material was not set up against a material of non-MM tumours relevant in differential diagnosis. Thus, our results on specificity of MM IHC markers cannot be generalized, although the results do reflect the historical development of MM IHC diagnostics.

For epithelial MM, IMP and IMIG recommend a combination of at least two positive and two negative markers, and a broad spectrum CK mix. They point out that the preferred markers depend on the experience of the laboratory, and that more mesothelial, epithelial, vascular, and malignant melanoma markers can become necessary if the results are not conclusive [6,23]. In our study, in the first IHC step, we followed these recommendations, except for the broad spectrum CK antibodies. However, we used three broad spectrum CK antibodies (AE1, AE3, KL1) together with other markers in some cases of possible sarcomatous MM where negative IR on these antibodies would indicate that the tumour was not a MM. For a diagnosis of epithelial and mixed MM, more than two positive and negative markers were considered being necessary in 12% and 25%, respectively.

Concerning the choice of IHC markers, Marchevsky and Wick point out the substantial value that odds rate calculations, systematic reviews and meta-analysis may have for the development of evidence-based guidelines for MM diagnosis [48]. This is even true if there are several possible applicable "optimal" marker combinations, dependant on the experience of the laboratory and the specific case.

A recent study on accuracy of pathological MM diagnosis based on a more than 5-fold larger Japanese material (382/73) set up as interdisciplinary re-evaluation shows with 15.8% (47/298) of all cases diagnosed by IHC a similar scale of diagnoses classified as "definitively not/unlikely" MM, compared with 11.5% (7/61) in our study [1]. The authors defined 80.5% (240/298) of the IHC cases as "probable/definite" MM, while the corresponding amount in our study was virtually the same, 80.3% (49/61). However, in the Japanese study, even cytological material and not furthermore processed histological slides were included; IHC was only performed in 80.6% (308/382) of the cases. As in our study, diagnostic accuracy was lower in sarcomatoid lesions. While there was no agreement in diagnoses in as much as 22.4% of all female pleural and 72.2% of all female peritoneal cases, the respective numbers in our study are 0% (0/6) and 50% (2/4).

Detailed data on exposition to asbestos or other possible MM risk factors were not available to us. By using census data from 1970 where occupations had been classified into having high, moderate or little/no asbestos exposure and expanding them to the 1960 and 1980 censuses, we have earlier made estimations on the possible asbestos exposure of nearly the same patient group (n = 47, two patients less than in the current study). Occupational asbestos exposure was likely in 12 patients, all men. However, these data could not be validated [11].

Survival analysis revealed no surprising data in respect of recent oncological knowledge on MM or malignant tumours in general. The presence of atypical mitoses alone, irrespective of the number of mitoses, could possibly be an independent negative prognostic factor, but this has to be verified or falsified on a larger material. Any sex preponderance in MM survival could not be seen in our material. The results of multivariate linear analysis may not be representative because of the too low number of cases for this purpose.

## Conclusions

Our results underline the need of histopathological re-evaluation of tissue biopsies with an earlier diagnosis of MM, especially those given before the introduction of at least one of the more specific positive MM markers that showed a good diagnostic reliability in our cases. In our opinion, a reliable IHC-based definition of the sarcomatous MM type has been absent for cases with negative IR for mesothelium-specific markers. We have considered such cases not being sufficient for a definitive pathological MM diagnosis, but if clinical and radiological findings support a MM diagnosis in such a setting, nevertheless, a MM diagnosis may be established. Differential diagnoses in our material were adenocarcinoma, pleomorphic and sarcomatoid carcinoma and sarcoma. The WHO/IMP and IMIG recommendations of at least two positive and two negative IHC markers can be affirmed by our study, as far as sensitivity is concerned. The marker choice has to be adapted to the specific differential diagnostic needs in each single case. In difficult cases, it may be useful to refer to a second opinion.

In six cases, despite of relatively extensive IHC application, we were not able to give a clear-cut entity diagnosis, which may highlight the need of novel diagnostic tools as biomarkers and other molecular analyses, as well as new criteria. Such new tools will also be needed for the more personalized approaches for cancer treatment in the future.

## Competing interests

The authors declare that they have no competing interests.

## Authors' contributions

HS participated in the design of the study, established histological diagnoses, processed the data and drafted the manuscript.

ODR participated in the design of the study and reviewed the manuscript.

KK provided data from the Cancer Registry of Norway and reviewed the manuscript.

HW established histological diagnoses and reviewed the manuscript.

EL participated in the design of the study, established histological diagnoses and reviewed the manuscript.

All authors read and approved the final manuscript.

## Supplementary Material

Additional file 1 **Comparison of primary and re-evaluation IHC/HC in the 12 cases where MM diagnosis was not confirmed, chronologically**. Click here for file
