# Supplementary material for: Re-evaluation of histological diagnoses of malignant mesothelioma by immunohistochemistry
Source: Diagn Pathol. 2010 Jul 6;5:47. doi: 10.1186/1746-1596-5-47 (PMC2915960; doi:10.1186/1746-1596-5-47)
Supplement: Additional file 1 — Comparison of primary and re-evaluation IHC/HC in the 12 cases where MM diagnosis was not confirmed, chronologically. [file 1746-1596-5-47-S1.DOC]

Additional file 1. Comparison of primary and re-evaluation IHC/HC in the 12 cases where MM diagnosis was not confirmed, chronologically

| Year of diagnosis | Patient no. | Positive markers | | | | | | | Negative markers | | | | | Other markers | | | | |
| --- | --- | --- | --- | --- | --- | --- | --- | --- | --- | --- | --- | --- | --- | --- | --- | --- | --- | --- |
|  |  | Calretinin nuclear | CK5/6 | EMA membranous | Podoplanin | Mesothelin | HBME-1 | Trombomodulin membranous | Sialyl-TN | CD15 | TTF-1 | CEA | Ber-Ep4 | CD34 | CD99 | Bcl-2 | CK AE1/AE3 | CK KL1 |
| **1986** | **5** | ⁄ | ⁄ | ⁄ | ⁄ | ⁄ | ⁄ | ⁄ | ⁄ | ⁄ | ⁄ | ⁄ | ⁄ | ⁄ | ⁄ | ⁄ | ⁄ | ⁄ |
| ***2007*** | **5** | **(+)** | **-** | **-** | **(+)** | **-** | **(+)** | **s +** | **-** | **-** | **-** | **-** | **-** | **-** | **(+)** | **-** | **s (+) e ++**  **11)** | **-** |
| **1988** | **3** | **⁄** | **⁄** | **⁄** | **⁄** | **⁄** | **⁄** | **⁄** | **⁄** | **⁄** | **⁄** | **⁄** | **⁄** | **⁄** | **⁄** | **⁄** | **⁄** | **⁄** |
| ***2007*** | **3** | **-** | **-** | **-**  **cp +** | - | **-** | (±) | **(+)** | **-** | **-** | **+** | **-** | **+** | ⁄ | ⁄ | ⁄ | ⁄ | ⁄ |
| **1988** | **6** | ⁄ | ⁄ | ⁄ | ⁄ | ⁄ | ⁄ | ⁄ | ⁄ | ⁄ | ⁄ | ⁄ | ⁄ | ⁄ | ⁄ | ⁄ | ⁄ | ⁄ |
| ***2007*** | **6** | **-** | **(+)** | **(+)**  **cp ++** | ⁄ | ⁄ | (±) | **-** | **-** | **-** | **+** | **-** | **-** | ⁄ | ⁄ | ⁄ | **s/e ++** | **+** |
| **1989** | **57** | ⁄ | ⁄ | ⁄ | ⁄ | ⁄ | ⁄ | ⁄ | ⁄ | ⁄ | ⁄ | ⁄ | ⁄ | ⁄ | ⁄ | ⁄ | ⁄ | ⁄  3)++ |
| ***2007*** | **57** | **(+)** | + | **-** | **-** | **-** | **(+)** | **-** | **-** | **-** | - | **-** | **-** | **-** | ⁄ | **(+)** | **++** | **++** |
| **1991** | **9** | ⁄ | ⁄ | ⁄ | ⁄ | ⁄ | ⁄ | ⁄ | ⁄ | ⁄ | ⁄ | ⁄ | ⁄ | ⁄ | ⁄ | ⁄ | ⁄ | ⁄ |
| ***2007*** | **9** | **-** | **-** | **(+)**  **cp ++** | **-** | **-** | **+** | **-** | **+** | **++** | **++** | **(+)** | **++** | ⁄ | ⁄ | ⁄ | ⁄ | ⁄ |
| **1996** | **72** | ⁄ | ⁄ | ⁄ | ⁄ | ⁄ | ⁄ | ⁄ | ⁄ | ⁄ | ⁄ | **?** | ⁄ | ⁄ | ⁄ | ⁄ | ⁄ | ⁄  10)  ++/+ |
| ***2007*** | **72** | **-** | (±) | **-**  **cp +** | **-** | **-** | **++** | **++** | **-** | **-** | **+** | **-** | **+** | ⁄ | ⁄ | ⁄ | ⁄ | ⁄ |
| **1998** | **31** | ⁄ | ⁄ | ⁄ | ⁄ | ⁄ | ⁄ | ⁄ | ⁄ | ⁄ | ⁄ | ⁄ | ⁄ | ⁄ | ⁄ | ⁄ | 1) ? | ⁄ |
| ***2007*** | **31** | **-** | **-** | **-** | **+** | **-** | **-** | **(+)** | **-** | **-** | **++** | **-** | **-** | **-** | **-** | **-** | **++** | **++** |
| **1998** | **49** | ⁄ | ⁄ | ⁄ | ⁄ | ⁄ | ⁄ | ⁄ | ⁄ | ⁄ | ⁄ | ⁄ | ⁄ | ⁄ | ⁄ | ⁄ | 1) + | ⁄ |
| ***2007*** | **49** | **-** | **-** | **-** | **-** | **-** | **-** | **-** | **-** | **-** | - | **-** | **-** | **+** | **-** | **+** | **+** | **+** |
| **1999** | **32** | **-** | ⁄ | **-** | ⁄ | ⁄ | ⁄ | ⁄ | ⁄ | ⁄ | ⁄ | **-** | **-** | **-** | ⁄ | ⁄ | 1) (±) | ⁄ |
| ***2007*** | **32** | **-** | **-** | **-** | **+** | **-** | **-** | **(+)** | **-** | **-** | **-** | **-** | **-** | **-** | **+** | **-** | **-** | **-** |
| **1999** | **58** | ⁄ | ⁄ | **cp ?**  **++** | ⁄ | ⁄ | ⁄ | ⁄ | ⁄ | ⁄ | ⁄ | ⁄ | **-** | ⁄ | ⁄ | ⁄ | 1) ++ | ⁄ |
| ***2007*** | **58** | **(+)** | **-** | **-**  **cp ++** | ⁄ | ⁄ | **++** | **(+)** | **+** | **-** | **-** | **-** | **+** | ⁄ | ⁄ | ⁄ | ⁄ | ⁄ |
| **2001** | **63** | **-** | ⁄ | **-** | ⁄ | ⁄ | **-** | ⁄ | ⁄ | ⁄ | ⁄ | ⁄ | **-** | ⁄ | ⁄ | ⁄ | ⁄ | ⁄  3)++ |
| ***2007*** | **63** | **-** | **-** | **-** | **-** | **-** | **-** | **-** | **-** | **-** | **-** | **-** | **-** | (?) | **(+)** | **-** | **++** | **++** |
| **2002** | **12** | **-** | ⁄ | **-** | ⁄ | ⁄ | ⁄ | ⁄ | ⁄ | ⁄ | ⁄ | - | **-** | ⁄ | ⁄ | ⁄ | ⁄ | ⁄ |
| ***2007*** | **12** | **-** | **-** | **-** | **+** | **(+)** | **-** | **-** | **-** | **-** | **-** | **-** | **-** | **-** | **-** | **-** | **++** | **++** |

| Year of diagnosis | Patient no. | Other markers | | | | | | | | | | | | | | | Our diagnosis |
| --- | --- | --- | --- | --- | --- | --- | --- | --- | --- | --- | --- | --- | --- | --- | --- | --- | --- |
|  |  | S-100 | HMB-45 | CD10 | CD68 | CD117 | Desmin | HHF-35 | SMA | Myoglobin | Vimentin | LCA | NSE | Others | MIB1 (% of MM cells) | Alcian blue |  |
| **1986** | **5** | ⁄ | ⁄ | ⁄ | ⁄ | ⁄ | ⁄ | ⁄ | ⁄ | ⁄ | ⁄ | ⁄ | ⁄ | ⁄ | ⁄ | ⁄ |  |
| ***2007*** | **5** | **-** | ⁄ | **+** | ⁄ | ⁄ | **-** | **-** | **-** | ⁄ | ⁄ | ⁄ | ⁄ | ⁄ | **s 25,**  **e less** | **-** | Undetermined: Sarcoma (not furthermore specified; leiomyosarcoma and malignant solitary fibrous tumor of the pleura excluded), sarcomatous MM possible, pleura/lung |
| **1988** | **3** | **⁄** | **⁄** | ⁄ | ⁄ | ⁄ | ⁄ | ⁄ | ⁄ | ⁄ | ⁄ | ⁄ | ⁄ | ⁄ | **⁄** | **⁄** |  |
| ***2007*** | **3** | ⁄ | **-** | ⁄ | ⁄ | ⁄ | ⁄ | ⁄ | ⁄ | ⁄ | ⁄ | ⁄ | ⁄ | ⁄ | ⁄ | ⁄ | AC, lung |
| **1988** | **6** | ⁄ | ⁄ | ⁄ | ⁄ | ⁄ | ⁄ | ⁄ | ⁄ | ⁄ | ⁄ | ⁄ | ⁄ | ⁄ | ⁄ | ⁄ |  |
| ***2007*** | **6** | ⁄ | ⁄ | ⁄ | ⁄ | ⁄ | ⁄ | ⁄ | ⁄ | ⁄ | ⁄ | ⁄ | ⁄ | ⁄ | **(+)** | **+** | Pleomorphic/mixed carcinoma of the lung consisting of components of sarcomatous carcinoma and adenocarcinoma |
| **1989** | **57** | ⁄ | ⁄ | ⁄ | ⁄ | ⁄ | ⁄ | ⁄ | ⁄ | ⁄ | ++ | ⁄ | ⁄ | 4) (*±*)  5) (*±*) | ⁄ | ⁄ |  |
| ***2007*** | **57** | **(+)** | ⁄ | **+** | ⁄ | ⁄ | **-** | **-** | **+** | ⁄ | ⁄ | ⁄ | ⁄ | ⁄ | **15** | **-** | Undetermined: sarcomatous MM more likely than sarcoma, chest wall/rib |
| **1991** | **9** | ⁄ | ⁄ | ⁄ | ⁄ | ⁄ | ⁄ | ⁄ | ⁄ | ⁄ | ⁄ | ⁄ | ⁄ | ⁄ | ⁄ | ⁄ |  |
| ***2007*** | **9** | **-** | **-** | (+) | ⁄ | ⁄ | - | ⁄ | ⁄ | ⁄ | ⁄ | ⁄ | ⁄ | ⁄ | ⁄ | ⁄ | Pleomorphic/giant cell carcinoma, lung |
| **1996** | **72** | ⁄ | ⁄ | ⁄ | ⁄ | ⁄ | ⁄ | ⁄ | ⁄ | ⁄ | ? | ⁄ | ⁄ | 9)? | ⁄ | ⁄ |  |
| ***2007*** | **72** | ⁄ | ⁄ | ⁄ | ⁄ | ⁄ | ⁄ | ⁄ | ⁄ | ⁄ | ⁄ | ⁄ | ⁄ | ⁄ | ⁄ | ⁄ | AC, lung |
| **1998** | **31** | ? | ? | ⁄ | ⁄ | ⁄ | ⁄ | ⁄ | ⁄ | ⁄ | ⁄ | ? | ? | ⁄ | ⁄ | ⁄ |  |
| ***2007*** | **31** | ⁄ | ⁄ | ++ | - |  | ⁄ | ⁄ | ⁄ | ⁄ | ⁄ | ⁄ | ⁄ | ⁄ | ⁄ | **-** | Pleomorphic carcinoma, lung |
| **1998** | **49** | **-** | ⁄ | ⁄ | - | ⁄ | - | ⁄ | - | - | ++ | - | ⁄ | ⁄ | ⁄ | ⁄2)+ |  |
| ***2007*** | **49** | **-** | ⁄ | **++** | ⁄ | **-** | ⁄ | ⁄ | **-** | ⁄ | ⁄ | ⁄ | ⁄ | ⁄ | **25** | **-** | Undetermined: sarcoma or sarcomatous carcinoma in pelvic peritoneum, ovarial tumor? |
| **1999** | **32** | ⁄ | ⁄ | ⁄ | ⁄ | ⁄ | - | ⁄ | ++ | ⁄ | ++ | ⁄ | ⁄ | ⁄ | ⁄ | ⁄ |  |
| ***2007*** | **32** | **-** | ⁄ | **++** | **++** | ⁄ | ⁄ | ⁄ | ⁄ | ⁄ | ⁄ | ⁄ | ⁄ | ⁄ | **20** | **-** | Undetermined: sarcoma, lung or pleura/sarcomatous MM, pleura |
| **1999** | **58** | ⁄ | ⁄ | ⁄ | ⁄ | ⁄ | ⁄ | ⁄ | ⁄ | ⁄ | ++ | ⁄ | ⁄ | 6) ++  7) - | ⁄ | ⁄ |  |
| ***2007*** | **58** | ⁄ | ⁄ | ⁄ | ⁄ | ⁄ | ⁄ | ⁄ | ⁄ | ⁄ | ⁄ | ⁄ | ⁄ | ⁄ | ⁄ | **-** | AC of peritoneum most likely |
| **2001** | **63** | ⁄ | ⁄ | ⁄ | ⁄ | ⁄ | ⁄ | ⁄ | ⁄ | ⁄ | ⁄ | ⁄ | ⁄ | 8) - | ⁄ | ⁄ |  |
| ***2007*** | **63** | ⁄ | ⁄ | + | ⁄ | ⁄ | ⁄ | ⁄ | ⁄ | ⁄ | ⁄ | ⁄ | ⁄ | ⁄ | ⁄ | **-** | Undetermined: sarcoma, lung or pleura/sarcomatous MM, pleura |
| **2002** | **12** | ⁄ | ⁄ | ⁄ | ⁄ | ⁄ | ⁄ | ⁄ | ⁄ | ⁄ | ++ | ⁄ | ⁄ | ⁄ | ⁄ | ⁄ |  |
| ***2007*** | **12** | (±) | **-** | **+** | ⁄ | ⁄ | ⁄ | ⁄ | ⁄ | ⁄ | ⁄ | ⁄ | ⁄ | ⁄ | **20** | ⁄ | Undetermined: AC, lung more likely than epithelial MM, pleura |

Year in italics= year of re-evaluation

IR:

⁄ not performed

(+) positive only in a few cells

+ focally positive

++ positive in most of tumour cells

- negative

(±) weakly positive in a few cells, uncertain IR

cp cytoplasmatic

e epithelial part

s sarcomatous part

? results unknown

(?) tumour is missing in deeper sections

1) pan-CK not specified

2) PAS with and without diastase

3) CK CAM5.2

4) alpha 1-antitrypsin

5) Factor VIII

6) CK7

7) CK20

8) LP-34

9) Chromogranin

10) low/high molecular weight CK

11) not sure whether the component is a part of the tumour
